# Interleukin-8 as a potential prognostic biomarker in renal cell carcinoma: a systematic review and meta-analysis

**DOI:** 10.1093/oncolo/oyaf254

**Published:** 2025-08-21

**Authors:** Francesca Zacchi, Sara Merler, Ilaria Zampiva, Irene Torresan, Stefano Manduca, Michela Piacentini, Clara Lorenzi, Emanuela Fantinel, Sarah Pafumi, Nicola Inzerilli, Anna Caliò, Matteo Brunelli, Alessandro Veccia, Alessandro Antonelli, Luca Tondulli, Fabiana Conciatori, Chiara Bazzichetto, Giuseppe Verlato, Lorena Torroni, Michele Milella

**Affiliations:** Section of Innovation Biomedicine—Oncology Area, Department of Engineering for Innovation Medicine, University of Verona and University and Hospital Trust (AOUI) of Verona, Verona 37134, Italy; Centro Ricerche Cliniche, Azienda Ospedaliera Integrata, Verona 37134, Italy; Section of Innovation Biomedicine—Oncology Area, Department of Engineering for Innovation Medicine, University of Verona and University and Hospital Trust (AOUI) of Verona, Verona 37134, Italy; Oncology Department, San Maurizio Central Hospital of Bolzano, South Tyrolean Health Service, Bolzano 39100, Italy; Section of Innovation Biomedicine—Oncology Area, Department of Engineering for Innovation Medicine, University of Verona and University and Hospital Trust (AOUI) of Verona, Verona 37134, Italy; Oncology Unit 1, Istituto Oncologico Veneto, IOV—IRCCS, Padova 35128, Italy; Section of Innovation Biomedicine—Oncology Area, Department of Engineering for Innovation Medicine, University of Verona and University and Hospital Trust (AOUI) of Verona, Verona 37134, Italy; Section of Innovation Biomedicine—Oncology Area, Department of Engineering for Innovation Medicine, University of Verona and University and Hospital Trust (AOUI) of Verona, Verona 37134, Italy; Section of Innovation Biomedicine—Oncology Area, Department of Engineering for Innovation Medicine, University of Verona and University and Hospital Trust (AOUI) of Verona, Verona 37134, Italy; Section of Innovation Biomedicine—Oncology Area, Department of Engineering for Innovation Medicine, University of Verona and University and Hospital Trust (AOUI) of Verona, Verona 37134, Italy; Section of Innovation Biomedicine—Oncology Area, Department of Engineering for Innovation Medicine, University of Verona and University and Hospital Trust (AOUI) of Verona, Verona 37134, Italy; Section of Innovation Biomedicine—Oncology Area, Department of Engineering for Innovation Medicine, University of Verona and University and Hospital Trust (AOUI) of Verona, Verona 37134, Italy; Department of Oncology, Ospedale Pederzoli, Verona 37019, Italy; Department of Diagnostic and Public Health, Section of Pathology, University of Verona, Verona 37134, Italy; Department of Diagnostic and Public Health, Section of Pathology, University of Verona, Verona 37134, Italy; Department of Urology, University of Verona, Verona 37126, Italy; Department of Urology, University of Verona, Verona 37126, Italy; Oncology Department, San Maurizio Central Hospital of Bolzano, South Tyrolean Health Service, Bolzano 39100, Italy; Preclinical Models and New Therapeutic Agents Unit, IRCCS Regina Elena National Cancer Institute, Rome 00144, Italy; Preclinical Models and New Therapeutic Agents Unit, IRCCS Regina Elena National Cancer Institute, Rome 00144, Italy; Unit of Epidemiology and Medical Statistics, Department of Diagnostics and Public Health, University of Verona, Verona 37134, Italy; Saint Camillus International, University of Health and Medical Sciences, Rome 00131, Italy; Section of Innovation Biomedicine—Oncology Area, Department of Engineering for Innovation Medicine, University of Verona and University and Hospital Trust (AOUI) of Verona, Verona 37134, Italy

**Keywords:** IL-8, renal cell carcinoma, immunotherapy, tyrosine kinase inhibitors drugs, meta-analysis

## Abstract

**Background and Objective:**

Interleukin-8 (IL-8) is a chemokine involved in inflammation and primary immune response, playing a key role in recruiting neutrophils. IL-8 is produced by several cell types, including immune cells and certain cancer cells. Elevated levels of IL-8 have been associated with a poorer outcome in several tumors and have been related to advanced diseases, treatment resistance, and possibly promoting neo angiogenesis and immune cell recruitment. In the renal cell carcinoma (RCC), the prognostic role of IL-8 has not been settled.

**Methods:**

From January 1, 2008 to June 18, 2024, PubMed, Embase, and Scopus databases were searched for all studies investigating the potential prognostic role of IL-8 in RCC. All studies were rated according to the Newcastle-Ottawa Scale. Progression-free survival (PFS) and overall survival (OS) were analyzed as clinical outcomes, and a meta-analysis was performed for both.

**Key Findings and Limitations:**

Overall, six papers met the predefined inclusion criteria, with final analyses demonstrating that high IL-8 levels significantly correlate with worse prognosis in RCC, with a statistically shorter PFS (hazard ratio [HR]: 1.27, 95% confidence interval [CI]: 1.01-1.59; *P* = 0.037) and statistically shorter OS (HR: 1.85, 95% CI: 1.21-2.84; *P* = .001). Prospective randomized clinical trials are also necessary to investigate the predictive role of IL-8 in the contemporary treatment scenario of RCC to improve clinical decision-making.

**Conclusions:**

This meta-analysis demonstrates the significant prognostic role of IL-8 in RCC, suggesting that IL-8 could be used to refine the prognostic RCC assessment.

Implications for Practice: Elevated circulating IL-8 levels are associated with worse prognosis in advanced RCC, supporting its role as a negative prognostic biomarker. This effect appears more pronounced in patients receiving immunotherapy or mTOR inhibitors, suggesting a potential role for IL-8 in treatment stratification. Prospective studies are needed to validate IL-8–guided therapeutic decision-making in contemporary RCC management.

## Introduction

Kidney cancer represents 3% of all diagnosed cancers, with an estimated worldwide incidence greater than 430 000 cases. Renal cell carcinoma (RCC) accounts for approximately 90%, with the most common histological subtype represented by clear cell RCC (ccRCC).^1^^,^^2^

Up to 30% of those initially diagnosed as localized tumors experience metastatic progression during follow-up, with lung, bone, and liver representing the most frequently involved sites. Moreover, about half of RCC patients are initially diagnosed with an advanced disease.^2^

The treatment landscape for RCC is constantly evolving and is currently based on immune checkpoint inhibitors and antiangiogenic targeted therapies. The decision-making process is largely based on risk stratification models (ie, Memorial Sloan-Kettering Cancer Center [MSKCC] and International Metastatic RCC Database Consortium [IMDC]), whose role is prognostic rather than predictive. Therefore, novel predictive biomarkers are needed to tailor individual treatment choices to disease biology rather than patients’ prognosis.

Interleukin-8 (IL-8, a.k.a. CXCL8) is a chemokine produced by both immune and non–immune cellular populations (ie, endothelial and cancer cells)^3–6^ and is normally undetectable in quiescent cells because of a three-step negative regulation mediated by NF-κb,^7^ OCT-1,^8^ and HDAC-1^9^ factors.

**Figure 1.**
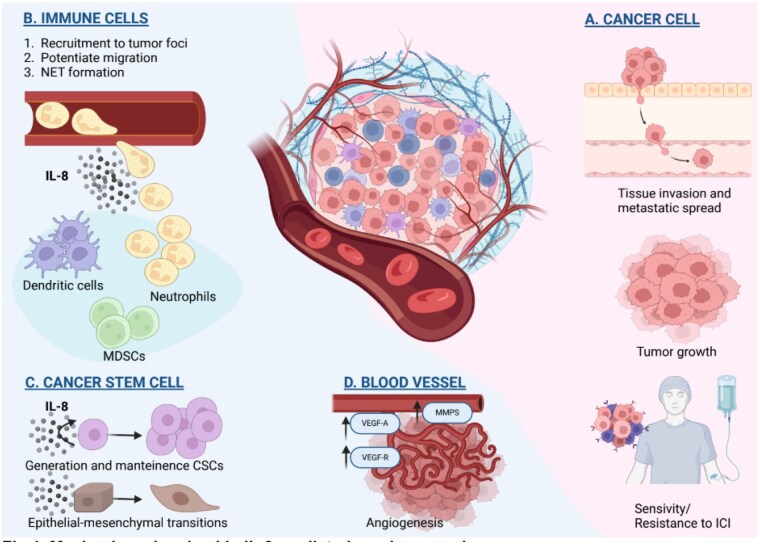
Mechanisms involved in IL-8-mediated carcinogenesis. IL-8 is released by cancer, immune, and stromal cells. This molecule promotes tumor progression and metastatic dissemination by acting on different kind of cells: cancer cells (A) and cells of tumor microenvironment (B–D) via autocrine and paracrine loops. (A) IL-8 acts directly on cancer cells, promoting progression and metastatic dissemination through various mechanisms: enhancing cell proliferation, avoiding stress-induced apoptosis, and promoting tissue migration and invasion. Its circulating and tissue levels also influence ICI sensitivity/resistance. (B–D) IL-8 remodels composition of tumor microenvironment. (B) IL-8 changes leucocyte infiltration, resulting in the accumulation of immunosuppressive and protumorigenic immune cells. It promotes the recruitment of neutrophils and MDSCs, which suppress anti-tumor immune responses (ie, through NET formation). (C) IL-8 supports CSCs proliferation and drives the epithelial-to-mesenchymal transition, which increases metastatic dissemination, stemness, and intrinsic resistance. (D) IL-8 promotes angiogenesis, enhancing the expression of MMPs, VEGF-R on endothelium, and release of VEGF-A. Abbreviations: CSCs, cancer stem cells; ICI, immune checkpoint inhibitor; IL-8, interleukin-8; MMPs, matrix metalloproteinases; NET, neutrophil extracellular trap; VEGF-A, vascular endothelial growth factor A; VEGF-R, vascular endothelial growth factor receptor. Image was created in BioRender. Lorenzi, C. (2025), https://BioRender.com/v59p408.

In the tumor microenvironment (TME), IL-8 signaling is involved in many steps of carcinogenesis and resistance to different therapies.^3^^,^^5^ It enhances tumor growth through increased cell proliferation and apoptosis evasion.^10^ Cancer cells can synthesize and secrete IL-8, acting in an autocrine and paracrine manner to self-maintain this altered proliferative loop.^3^^,^^11^^,^^12^ Moreover, IL-8 is also involved in neo-angiogenesis, enhancing the expression of metalloproteinases (MMPs) that disrupt stromal structures and create vascular lacunae.^13^ In addition, literature data support the presence of an interplay between IL-8 and the vascular endothelial growth factor (VEGF)/ vascular endothelial growth factor receptor (VEGF-R) axis that unfolds in increased VEGF-A release and endothelial expression of VEGF-R.^14^^,^^15^ Other mechanisms directly involved in IL-8-mediated carcinogenesis include cancer stem cells (CSCs) maintenance,^16^ metabolic glycolytic shift,^17^ epithelial to mesenchymal transition (EMT),^18^ and TME reprogramming.^19^^,^^20^ At the tumor/host interface, IL-8 is especially involved in neutrophil recruitment, in physiological and pathological conditions.^3^^,^^4^ It has been demonstrated that the creation of neutrophil extracellular traps (NETs) enhances cancer cells’ local invasiveness.^21^^,^^22^ In addition to neutrophils, IL-8 recruits other immune cells, such as tumor-associated macrophages (TAMs) and myeloid-derived suppressor cells (MDSCs),^11^^,^^22^ that may actively reduce immune response in TME (Figure 1).

In RCC, high IL-8 expression has been associated with a higher tumor burden and, potentially, worse prognosis.^23^ Furthermore, many data support a role for IL-8 in allowing RCC to escape immune-mediated suppression and restoring angiogenesis, despite VEGF/VEGF-R axis blockade.^24–26^ Thus, IL-8 likely represents a potential prognostic/predictive biomarker due to its pro-inflammatory, immune-suppressive, and pro-angiogenic activity.^3^^,^^4^ However, its significance and impact in RCC prognosis have not been formally proven.

The present meta-analysis was conducted to establish a correlation between circulating or tissue IL-8 and clinical RCC outcomes, defined as overall survival (OS, primary endpoint) and progression-free survival (PFS, secondary endpoint) by evaluating published clinical studies on this topic, available as of June 2024.

## Evidence acquisition

### Search strategy

A systematic literature search was conducted using electronic databases (PubMed, Embase, and Scopus), including all publications between January 1, 2008, and June 18, 2024. The search strategy was designed by three authors (M.M, I.Z., and S.M.) and approved by all the other scientists, combining the following medical subject headings (MeSH) terms: “interleukin-8” or “IL-8” or “CXCL-8” or “CXCL8”, and “carcinoma” or “RCC” or “cancer” or “ccRCC” or “chRCC” or “pRCC”. Boolean operators were used to connect specific keywords (see Table S1. for the complete search strategy).

**Table 1.** The main characteristics of the papers included in the final meta-analysis: title, first author, year of publication, study design, histology and RCC subtypes, IMDC/MSKCC scores, RCC stage, and treatment.

Title	Authors	Year	IL-8 analysis	Histology and RCC subtypes	Stage	IMDC or MSKCC score	Type and line of treatment
**Hypertension and circulating cytokines and angiogenic factors in patients with advanced non-clear cell renal cell carcinoma treated with sunitinib: results from a phase II trial** ^27^	Bilen et al.	2015	Prospective	nccRCC (100%):• pRCC(47%), unclassified (14%),• sarcomatoid features (12%),• collecting duct/renal medullary (11%),• chRCC (9%),• other nccRCC (7%)	IV	MSKCC, 16% F, 63% I, 21% P	Sunitinib (maximum of two prior systemic therapies, and no prior treatment with TKIs)
**Plasma cytokine and angiogenic factors associated with prognosis and therapeutic response to sunitinib versus everolimus in advanced non-clear cell renal cell carcinoma** ^28^	Msaouel et al.	2017	Retrospective	ccRCC with sarcomatoid features (30%), pRCC (30%), chRCC (16%), RCC with translocation (8%), unclassified (16%)	IV	IMDC, 8% F, 81% I, 11% P	1L sunitinib versus EVE
**High systemic and tumor-associated IL-8 correlates with reduced clinical benefit of PD-L1 blockade** ^29^	Yuen et al.	2020	Retrospective	ccRCC (100%)	IV	MSKCC, 22% F, 69% I, 9% P	1L Atezo versus Atezo + BEV versus sunitinib
**Circulating proteins as potential biomarkers of sunitinib and interferon‑α efficacy in treatment‑naïve patients with metastatic renal cell carcinoma** ^30^	Harmon et al.	2013	Prospective	ccRCC (100%)	IV	MSKCC, 33% F, 67% I, 0% P	1L sunitinib versus IFN-α
**Elevated serum interleukin-8 is associated with enhanced intra-tumor neutrophils and reduced clinical benefit of immune-checkpoint inhibitors** ^31^	Schalper et al.	2020	Retrospective	ccRCC (100%)	Advanced or IV	MSKCC, 35% F, 49% I, 16% P	Nivolumab versus EVE (after one or two antiangiogenic therapy)
**Outcomes based on plasma biomarkers in METEOR, a randomized phase 3 trial of cabozantinib versus everolimus in advanced renal cell carcinoma** ^32^	Powles et al.	2021	Prospective	ccRCC (100%)	Advanced or IV	IMDC, 19% F, 64% I, 16% P	Cabozantinib versus EVE (after one or more VEGF-R TKIs)

Abbreviations: 1L, first line; BEV, bevacizumab; ccRCC, clear cell renal cell carcinoma; chRCC, chromophobe renal cell carcinoma; EVE, everolimus; F, favorable risk; I, intermediate risk; IFN-α, interferon alpha; IMDC, International Metastatic renal cell carcinoma Database Consortium; MSKCC, Memorial Sloan-Kettering Cancer Center; nccRCC, non-clear cell renal cell carcinoma; P, poor risk; pRCC, papillary renal cell carcinoma; TKI, tyrosine-kinase inhibitor.

The research investigated the potential prognostic role of IL-8 in RCC, and both OS (primary endpoint) and PFS (secondary endpoint) were analyzed as clinical outcomes. In addition, the correlation between IL-8 and DFS, as well as objective response rate (ORR), was also explored as secondary endpoints. However, no data were found regarding the correlation between IL-8 and DFS or ORR.

The systematic literature review and meta-analysis followed the preferred reporting items for systematic reviews and meta-analyses (PRISMA) guidelines (Figures S1 and S2, see online supplementary material for a color version of this figure). The review protocol was registered in the International Prospective Register of Systematic Reviews (PROSPERO ID: CRD42022297310).

**Figure 2.**
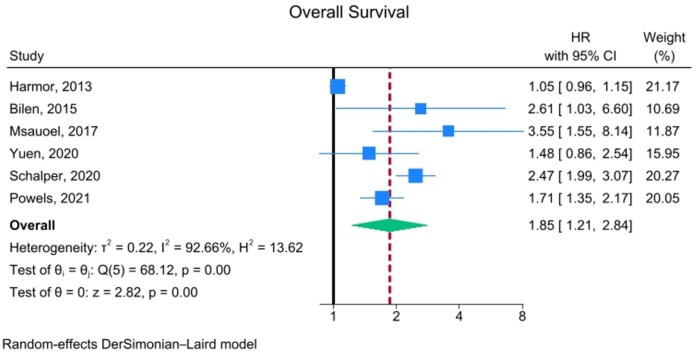
Studies investigating the role of circulating IL-8 in relation to OS in RCC. In this forest plot, we included all studies assessing OS with hazard ratio (HR) data derived from multi-variable analyses. Specifically, for the studies by Yuen, Harmor, Shalper, and Powels, which reported HRs for multiple treatment arms, “pooled” HRs were calculated to represent the average risk within each study for survival outcomes. For the study by Yuen, we reported the circulating IL-8 HR analysis. Abbreviations: HR, hazard ratio; IL-8, interleukin-8; OS, overall survival; RCC, renal cell carcinoma.

### Inclusion and exclusion criteria

The articles found with the search strategy have been screened for duplicate papers and selected by title, abstract, and full text according to the inclusion/exclusion criteria.

The inclusion criteria were as follows:

1. Randomized and non-randomized clinical trials.
2. IL-8 levels must be reported as correlated with at least one of the following outcome measures: OS or PFS.
3. The hazard ratio (HR) and the upper and lower limits of the confidence interval (CI) must be reported for the outcome, which must be 95% (95% CI).
4. If HR is not present, Kaplan–Meier or medians and number of events must be present.
5. All IL-8 expression evaluations, including serum, plasma, and tissue.

The exclusion criteria were as follows:

1. Papers reporting data that cannot be extracted.
2. Non–English papers.
3. Non–full-length papers.
4. Reviews, systematic reviews, meta-analyses, book chapters, and case reports.
5. Animal studies or basic research papers.
6. Studies on gene polymorphisms.
7. Studies not including RCC tumors.
8. Papers not relevant to the topic.

### Outcome of interest and quality assessment

Four authors (N.I., S.P., E.F., and I.Z.) collected and checked the data independently, and all data were revised by two independent reviewers (S.M. and F.Z.). Any disagreement was solved by another reviewer (M.M.). These study characteristics were collected for the review: title, first author, year of publication, country, study design, histology, tumor stage, method of IL-8 detection (circulating or tissue; IL-8 cut-off), number of patients (total number of patients with low IL-8 and patients with high IL-8), clinical endpoints (OS, PFS, with HR, 95% CI, *P*-value) follow-up, and treatments.

Observational studies were evaluated with the Newcastle-Ottawa Scale (NOS) to assess the risk of bias. This scale is commonly used to assess the quality of medical research in cohort/case-control studies. In detail, the NOS consists of an eight-point scale. The NOS for cohort studies assesses the categories selection, comparability, and outcome. For the categories selection and outcome, one star is awarded for each fulfilled item, while for comparability, a maximum of two stars can be awarded. In the meta-analysis, we only included studies with a good (NOS: 7-8) or excellent (NOS: 9) score.

### Statistical analysis

A meta-analysis was conducted to compare the results of the selected studies.

The HR was calculated for time-dependent variables.

To assess variability among studies, we performed a heterogeneity test and the *I*^2^ statistic, which quantifies the proportion of total variation in effect estimates due to heterogeneity rather than sampling error. When the heterogeneity test was not significant (*P* > 0.050), *I*^2^ was less than 30%, and significant heterogeneity was ruled out. In such case, a fixed-effects model was adopted to evaluate the pool of results using Mantel and Haenszel’s method. Otherwise, a random effects model was used, pooling results using the DerSimonian and Laird methods.

A subgroup analysis was conducted to explore potential sources of heterogeneity, stratifying by IL-8 analysis type (prospective vs. retrospective). Based on the treatment type, sensitivity analysis was performed to assess the robustness of the results.

The results were shown using forest plots.

All analyses were performed using STATA software, version 18 (StataCorp).

## Evidence synthesis

### Search results and included trials

The systematic literature review identified 2527 studies matching the specified keywords across three databases (PubMed, Embase, and Scopus). Following PRISMA guidelines (Figure S1, see online supplementary material for a color version of this figure), 784 duplicate studies were excluded from the initial screening. An additional 1736 studies were excluded for not meeting the inclusion and exclusion criteria, leaving only seven studies that met the pre-defined inclusion and exclusion criteria for the meta-analysis (PRISMA flowchart—Figure S3, see online supplementary material for a color version of this figure). Their quality was evaluated using the NOS, resulting in six final studies with good or excellent scores (NOS ≥ 7) (Tables S2 and S3).

**Figure 3.**
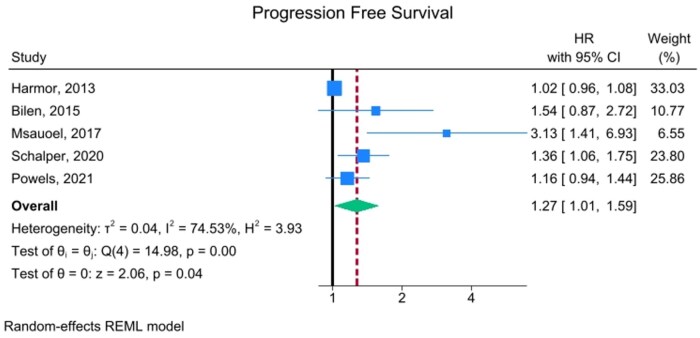
Studies investigating the role of circulating IL-8 in relation to PFS in RCC. This forest plot includes all studies on PFS with HR data derived from multi-variable analyses except for the study by Bilen, which evaluated data derived from univariable analysis. Specifically, for the Powel’s study, which reported HR for multiple treatment arms, a “pooled” HR was calculated to represent the average risk for survival outcomes within each study. Abbreviations: HR, hazard ratio; IL-8, interleukin-8; PFS, progression-free survival; RCC, renal cell carcinoma.

**Table 2.** Key characteristics of the papers included in the final meta-analysis: IL-8 detection, total number of patients, IL-8 cut-off, number of patients with low IL-8 levels, number with high IL-8 levels, OS, and PFS.

Title	Authors	IL-8 detection	Total number of patients	IL-8 cut-off	Patients with IL-8 (low)	Patients with IL-8 (high)	HR OS (95% CI)	HR PFS (95% CI)
**Hypertension and circulating cytokines and angiogenic factors in patients with advanced non-clear cell renal cell carcinoma treated with sunitinib: results from a phase II trial** ^27^	Bilen et al.	Circulating	53 RCC	Baseline median IL-8 levels: (22.32 pg/mL)	27	26	HR: 2.61^a^ 95% CI: 1.03-6.58	HR: 1.54^b^ 95% CI: 0.87-2.72
**Plasma cytokine and angiogenic factors associated with prognosis and therapeutic response to sunitinib versus everolimus in advanced non-clear cell renal cell carcinoma** ^28^	Msaouel et al.	Circulating	37 RCC, 16 EVE, 21 sunitinib	Baseline median IL-8 levels: 5.18 pg/mL (3.93-7.82) EVE, 3.70 pg/mL (2.74-5.89) sunitinib.	-	-	HR 3.55^a^ 95% CI: 1.55-8.14	HR 3.13^a^ 95% CI: 1.41-6.92
**High systemic and tumor-associated IL-8 correlates with reduced clinical benefit of PD-L1 blockade** ^29^	Yuen et al.	Circulating and tissue	248 RCC	Median IL-8 levels	124	124	Circulating IL-8 HR 2.55^a^ ^,^ ^c^; 95% CI: 1.8-5.5 (ATEZO); HR 1.25,^a^ ^,^ ^c^ 95% CI: 0.61-2.60, (ATEZO + BEV); HR 1.48,^a^ ^,^ ^c^ 95% CI: 0.69-3.2. (sunitinib).	NA
**Circulating proteins as potential biomarkers of sunitinib and interferon‑αefficacy in treatment‑naïve patients with metastatic renal cell carcinoma** ^30^	Harmon et al.	Circulating	63 RCC, 33 sunitinib, 30 IFN-α	Median baseline IL-8 levels: 7 pg/mL sunitinib 9.5 pg/mL IFN-α	-	-	HR 1.11,^a^ 95% CI: 1.022-1.2 (sunitinib) HR 1.013,^a^ 95% CI: 0.969-1.06; (IFN-α)	HR 1.018,^a^ 95% CI: 0.964-1.08; (IFN-α)
**Elevated serum interleukin-8 is associated with enhanced intra-tumor neutrophils and reduced clinical benefit of immune-checkpoint inhibitors** ^31^	Schalper et al.	Circulating	740 RCC, 392 nivolumab, 348 EVE	Baseline IL-8 levels (23 pg/mL)	269 Nivolumab, 241 everolimus	123 Nivolumab, 107 everolimus	HR: 2,56,^a^ 95% CI: 1,89-3,45, (nivolumab) HR: 2,4,^a^ 95% CI: 1,78-3,2, (everolimus)	HR: 1.36,^a^ 95% CI: 1,07-1.772 (nivolumab)
**Outcomes based on plasma biomarkers in METEOR, a randomized phase 3 trial of cabozantinib versus everolimus in advanced renal cell carcinoma** ^32^	Powles et al.	Circulating	621 RCC, 316 CABO, 305 EVE	Baseline median IL-8: 4.601 pg/mL CABO 5.049 pg/mL EVE	292	291	HR 1.77,^a^ 95% CI: 1.25-2.5 (CABO); HR 1.67,^a^ 95% CI: 1.23-2.27. (EVE)	HR 1.03^a^ 95% CI: 0.76-1.4 (CABO); HR 1.33^a^ 95% CI: 1.01-1.76 (EVE)

In all cases, the HR was assessed by comparing patients with high IL-8 levels to those with low IL-8 levels. Abbreviations: ATEZO, atezolizumab; BEV, bevacizumab; CABO, cabozantinib; CI, confidence interval; EVE, everolimus; HR, hazard ratio; IFN-α, Interferon alpha; OS, overall survival; PFS, progression-free survival.

a HR from multi-variate analysis.

b HR from univariate analysis.

c HR values related to plasmatic IL-8.

All six studies were retrospective or prospective cohort studies focusing on patients with advanced or metastatic RCC.^27–32^ Four studies examined ccRCC,^29–32^ and two also included non-ccRCC (nccRCC)^27^^,^^28^; very few patients with sarcomatoid histological features were represented. In all but one study IL-8 was measured in blood samples^27^^,^^28^^,^^30–32^; in the remaining study, IL-8 was evaluated in both blood and tissue samples.^29^ Characteristics of the studies included are summarized in Tables 1 and 2.

### Impact of IL-8 levels on OS and PFS of advanced RCC patients

Of these six studies, all were evaluated for the OS endpoint (1762 patients),^27–32^ and five were assessed for the PFS endpoint (1514 patients).^27^^,^^28^^,^^30–32^

High levels of circulating IL-8 were associated with significantly worse OS (HR: 1.85, 95% CI: 1.21-2.84, *P* = .001). Despite using a random effects model to account for variability among studies, the overall heterogeneity was high (*I*^2^ = 92.66%), suggesting that methodological differences, characteristics of the included patients, or other variables could influence the results (Figure 2).

The impact of IL-8 on the secondary endpoint of PFS was more limited but still significant, with high levels correlating with a worse outcome (HR: 1.27, 95% CI: 1.01-1.59, *P* = .037) (Figure 3). Heterogeneity is moderately high (*I*^2^ = 74.53%) among studies, although they are relatively consistent.

After excluding the only study based on univariate PFS analysis,^27^ the results still indicate a trend toward worse PFS in patients with high IL-8 levels, though the association remains non-significant (Figure S4, see online supplementary material for a color version of this figure).

**Figure 4.**
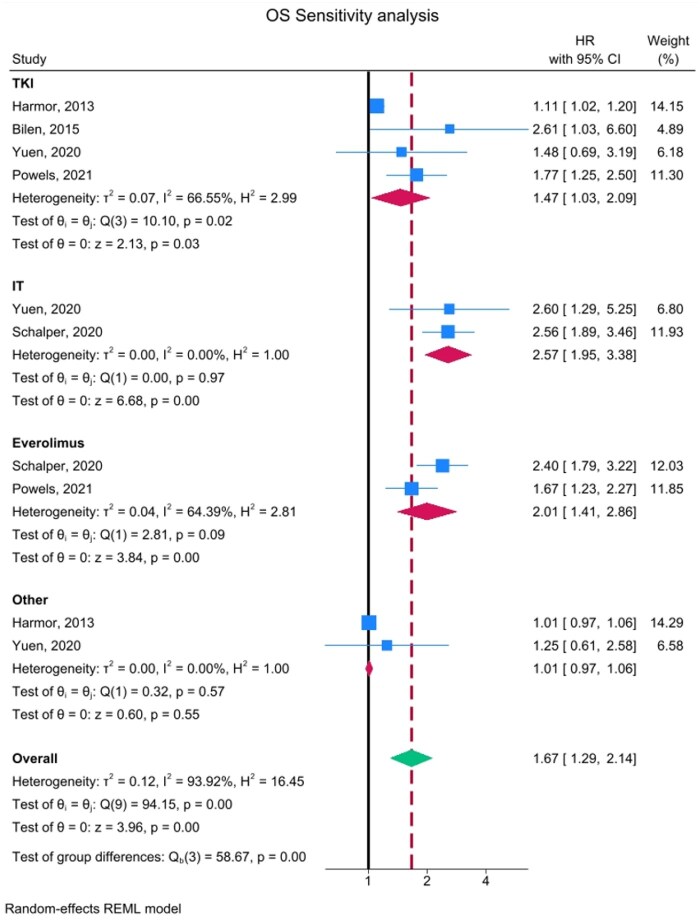
Sensitivity analysis. Forest plot of HR comparing OS in low and overexpressed IL-8 patients divided by therapy. HRs for each trial are represented by the squares, and the horizontal line crossing the squares represents the 95% CI. The diamonds represent the estimated overall effect based on the meta-analysis random effects of the trials. All statistical tests were two-sided. Abbreviations: CI, confidence interval; HR, hazard ratio; IL-8, interleukin-8; OS, overall survival.

Differences between retrospective and prospective studies were also analyzed separately: elevated levels of IL-8 were associated with a worse outcome of OS in retrospective studies (HR: 2.26, 95% CI: 1.53-3.35) and in prospective studies (HR: 1.47, 95% CI: 0.93-2.33). Prospective studies show greater heterogeneity (*I*^2^ = 88.41%) (Figure S5, see online supplementary material for a color version of this figure).

No data were found regarding the correlation between IL-8 and DFS or ORR.

Publication bias: The publication bias was evaluated with the funnel plot and Egger’s test. No publication bias was found (*P* = .067) in the survival outcomes. The Eggers’ test and the funnel plot are shown in Figure S6 (see online supplementary material for a color version of this figure).

### Sensitivity analysis

A sensitivity analysis for OS was conducted based on treatment type. OS analysis included all six studies and 10 different cohorts: four treated with tyrosine-kinase inhibitors (TKIs) such as cabozantinib or sunitinib, two with immunotherapy alone (atezolizumab or nivolumab), two with everolimus, and two with combinations or other therapies (atezolizumab + bevacizumab or IFN-α).

High circulating IL-8 had the strongest association with worse OS in cohorts treated with immunotherapy (HR = 2.57, 95% CI: 1.95-3.38) and everolimus (HR = 2.01, 95% CI: 1.41-2.86). A weaker effect but still significant was observed in TKI-treated cohorts (HR = 1.47, 95% CI: 1.03-2.09), while no difference was found for other therapies (HR = 1.01, 95% CI: 0.97-1.06) (Figure 4).

### TCGA-based expression analyses in RCC RNA-sequencing data resources

#### IL-8 expression analysis in RCC RNA-sequencing data resources

To validate the impact of IL-8 expression on RCC outcomes, we analyzed publicly available gene expression datasets^33–35^ derived from RNA sequencing, including data from large-scale genomic initiatives such as The Cancer Genome Atlas (TCGA). In resected ccRCC, high transcript-level expression of the *IL-8* gene evaluated in surgical tissue was significantly (*P* = .001) associated with worse OS in the Firehose Legacy dataset and showed a non-significant trend toward association with worse OS in the TCGA dataset (TCGA Nature 2013); no significant impact on disease-free survival (DFS) was observed in the only dataset in which data on this outcome were available (Firehose Legacy); (Figure S7, see online supplementary material for a color version of this figure). Conversely, in the same two datasets, analysis of nccRCC cases revealed a correlation of borderline significance (*P* = .05) between high transcript-level expression of the IL-8 gene evaluated in surgical tissue and worse DFS in the Firehose Legacy dataset (Figure S8, see online supplementary material for a color version of this figure).

#### IL-8 expression stratified by VHL mutation status across RNA-sequencing data resources

The relevance of VEGF signaling in the pathogenesis of clear cell RCC is well established and is underscored by the high prevalence of loss-of-function mutations in the von Hippel–Lindau (VHL) gene. The VHL protein plays a critical role in regulating angiogenesis by targeting the hypoxia-inducible factor (HIF) for degradation under normoxic conditions. When VHL is mutated or inactivated, HIF accumulates and drives the transcriptional upregulation of VEGF and other pro-angiogenic factors, thereby promoting tumor vascularization and progression.^36^

To further investigate whether VHL inactivation contributes to IL-8 upregulation in RCC, we examined the potential association between IL-8 expression levels and the genetic status of VHL through cBioportal (www.cbioportal.org). Specifically, we stratified TCGA RCC samples by VHL mutation status and evaluated IL-8 expression across subgroups.

In the ccRCC subgroup, IL-8 high and low expression did not segregate significantly according to VHL mutation status. However, we observed a non-significant trend toward enrichment of VHL mutations in IL-8 low tumors, suggesting that VHL alterations do not directly account for elevated IL-8 levels in this context (Figure S9, see online supplementary material for a color version of this figure).

In contrast, among nccRCC cases, VHL mutations were exceedingly rare, precluding any meaningful comparative analysis (Figure S10, see online supplementary material for a color version of this figure).

These findings suggest that while VHL mutations are common in ccRCC, they are not the primary determinant of IL-8 expression levels, and that IL-8 upregulation may occur independently of VHL mutation.

### Discussion

The findings reported herein demonstrate that elevated circulating IL-8 levels are associated with significantly worse outcomes in advanced RCC, particularly in terms of OS. Such association was relatively weaker for PFS and did not appear to depend on histological subtypes (ccRCC versus nccRCC). However, nccRCC was specifically investigated in only one study^27^ and was mixed with sarcomatoid ccRCC, translocation RCC, and unclassified RCC in another^28^; thus, the issue of a differential impact of high circulating IL-8 levels in nccRCC cannot be solidly addressed and remains a potential limitation of this meta-analysis, also considering the high degree heterogeneity of distinct histological subtypes included in the nccRCC subgroup. Moreover, in resected RCC tumor tissue high levels of IL-8 production (conveying a higher risk of death) did not appear to correlate with VHL mutational status. The VHL protein plays a critical role in regulating angiogenesis by targeting the HIF for degradation under normoxic conditions. When VHL is mutated or inactivated, HIF accumulates and drives the transcriptional upregulation of VEGF and other pro-angiogenic factors, thereby promoting tumor vascularization and progression.^36^ Although a mechanistic correlation between loss of VHL function, transcriptional HIF-1a activation, and IL-8 expression has been hypothesized, several lines of evidence indicate that IL-8 may constitute a HIF-independent, compensatory mechanism of response to hypoxia,^37^ together with evidence that basal and hypoxia-stimulated IL-8 levels do not correlate with *VHL* mutational status in RCC cell lines and cell-line derived spheres our findings suggest that *VHL* mutations are not the primary determinant of IL-8 expression levels in RCC, and that IL-8 upregulation may occur as an independent, compensatory event.^38^^,^^39^

The source from which IL-8 was assessed (circulating vs. tissue) does not appear to influence the results. Indeed, while the forest plot reported in Figure 2 refers to circulating IL-8 levels (high vs. low), in the only trial in which IL-8 was also assessed intra-tumorally at the tissue level,^29^ high IL-8 levels were similarly linked to worse outcomes (OS HR: 3.9, 95% CI: 2.8-5.5 in the atezolizumab arm; HR: 1.78, 95% CI: 0.61-2.60 in the atezolizumab + bevacizumab arm; HR: 1.75, 95% CI: 0.69-3.2 in the sunitinib arm). In addition, data available in public datasets regarding IL-8 transcript levels in tumor tissue show a qualitatively similar trend toward association with poorer survival outcomes in resected ccRCC and nccRCC alike, again suggesting that the prognostic/predictive impact of IL-8 is independent from either the source of assessment or the histological subtype. No statistically significant differences were observed considering the retrospective versus prospective design of the studies analyzed; however, the impact of IL-8 levels on advanced RCC appeared to be qualitatively more evident in retrospective studies, likely due to greater heterogeneity between studies in terms of patients’ populations and treatments in prospective trials.

Elevated IL-8 levels have been associated with poorer outcomes in other cancers, including non–small cell and small-cell lung cancer (NSCLC and SCLC, respectively),^31^^,^^40^ colorectal cancer (CRC),^41^ metastatic urothelial carcinoma,^29^ melanoma^31^ and metastatic prostate cancer (PCa). In metastatic PCa, elevated serum IL-8 is linked to a shorter time to castration-resistant disease and worse OS.^42^ Notably, Maynard et al. demonstrated that IL-8 expression in the TME correlates with aggressive prostate cancer and androgen receptor (AR) loss in metastatic disease.^43^^,^^44^ Previous meta-analyses conducted by our group on the prognostic/predictive impact of IL-8 in NSCLC and CRC^40^^,^^41^ align with the findings of this study, demonstrating a stronger impact of IL-8 on OS compared to PFS. This highlights the potential limitations of PFS as a surrogate for OS across different treatments and cancer subtypes,^44^^,^^45^ on the one hand, and suggests a prognostic rather than predictive effect for IL-8 on the other.

Sensitivity analyses conducted according to the different treatment cohorts assessed in the studies considered suggest a somewhat differential effect of IL-8 on survival outcomes, with the strongest impact observed in subgroups of patients treated with immunotherapy (HR: 2.57, 95% CI: 1.84-3.29) and the mTOR inhibitor everolimus (HR: 1.99, 95% CI: 1.28-2.70), a weaker impact on patients treated with TKIs (HR: 1.40, 95% CI: 0.90-1.90), and no effect in patients treated with other therapies. Similar data were reported in individual studies by Yuen et al.^29^ and Schalper et al.,^31^ showing a significant detrimental effect of high IL-8 circulating levels only in cohorts of RCC patients receiving single-agent atezolizumab, nivolumab, or everolimus, but not a combination of atezolizumab and bevacizumab or sunitinib alone. In our previous meta-analysis conducted in CRC, the impact of IL-8 on OS appeared to be slightly more evident in subgroups of patients treated with anti-angiogenic agents as compared with chemotherapy^46^; nevertheless, the benefit of adding anti-angiogenic agents to standard treatment or placebo in individual studies did not differ between high and low circulating IL-8 levels.^47^ In our previous meta-analysis conducted in lung cancer the impact of IL-8 expression on survival outcomes was more pronounced in subgroups treated with chemotherapy, slightly lower in subgroups treated with immunotherapy and almost undetectable in subgroups receiving other treatments (chemotherapy/immunotherapy/IT or targeted therapy^40^). Similar effects in cohorts of patients treated with either immunotherapy or chemotherapy were observed in squamous and non-squamous NSCLC, as well as in metastatic urothelial carcinoma.^29^^,^^31^ Moreover, in some of these studies,^40^ as well as in the cohorts of resected cc/nccRCC extracted from public databases and reported herein, high circulating IL-8 levels in patients treated only with surgery also showed a significant detrimental impact. Overall, available data suggest that IL-8 has a relevant negative prognostic impact in a wide range of human malignancies; such impact may also have predictive value in the context of chemotherapy or immunotherapy treatments, while it is much less relevant in the context of treatment with anti-angiogenic agents, targeted therapy (with the notable exception of mTOR inhibition in advanced RCC), and combined therapies (anti-angiogenesis/immunotherapy/IT, chemotherapy/immunotherapy/IT).

This is not surprising, considering the pro-angiogenic and pro-inflammatory role of the IL-8 chemokine^48^^,^^49^ which is highly expressed in RCC, a tumor type characterized by angiogenesis, immune infiltration, and an inflamed TME.^50^ In particular, IL-8 plays a critical role in recruiting immunosuppressive myeloid cells, such as MDSCs, into the TME. Najjar et al.^48^ demonstrated a significant correlation between elevated parenchymal IL-8 levels and increased MDSC infiltration in RCC patients undergoing nephrectomy,^19^ potentially resulting in the observed impaired efficacy of immunotherapy. It is interesting to note that elevated IL-8 levels do not seem to impact the outcomes of RCC patients treated with a combination of immunotherapy and anti-angiogenic agents.^29^ In the current RCC therapeutic scenario, immunotherapy IT/TKI combinations may thus override the negative prognostic impact of high IL-8 levels, thereby raising the intriguing possibility that IL-8 levels help choose between immunotherapy/TKI and immunotherapy combinations. No data with contemporary first-line combinations are currently available, calling for prospective studies, such as the SIGNS-RCC observational study promoted by our research group, to support this hypothesis.

## Conclusions

In conclusion, evidence derived from six prospective and retrospective cohort studies substantiates the prognostic significance of IL-8 in advanced RCC, in terms of both OS and, to a lesser extent, PFS; such prognostic effect is particularly evident in cohorts of patients treated with either immunotherapy or mTOR inhibitors. Furthermore, analyses of public transcriptomic databases suggest that such prognostic effects of IL-8 could also be applicable to surgically treated patients. Given the unique behavior of IL-8 in RCC TME^51^ and the usually poor correlation between circulating and tissue cytokine levels, investigating the source of IL-8 within RCC TME could offer further valuable insights (as also suggested by Yuen et al.^29^), potentially enhancing our understanding of tumor progression and therapeutic responses.

## Supplementary Material

oyaf254_Supplementary_Data

## Data Availability

Not applicable.
