# Supplementary material for: Interleukin-8 as a potential prognostic biomarker in renal cell carcinoma: a systematic review and meta-analysis
Source: Oncologist. 2025 Aug 21;30(9):oyaf254. doi: 10.1093/oncolo/oyaf254 (PMC12448429; doi:10.1093/oncolo/oyaf254)
Supplement: oyaf254_Supplementary_Data [file oyaf254_supplementary_data.zip › Supplementary Material Figures and Tables 14.07.25 - Copia.docx]

**Supplementary Figures, Tables and Supplementary References.**

**
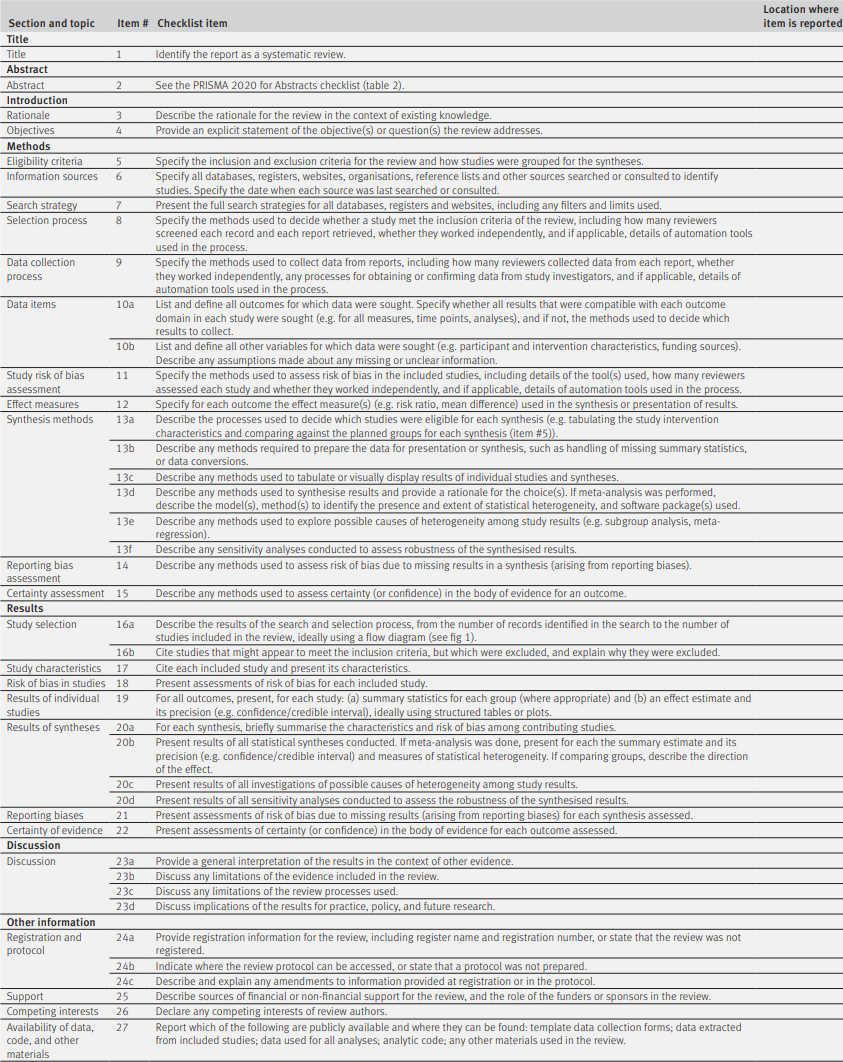
Supplementary Fig. S1. PRISMA 2020 item checklist** ^1^**.**

**
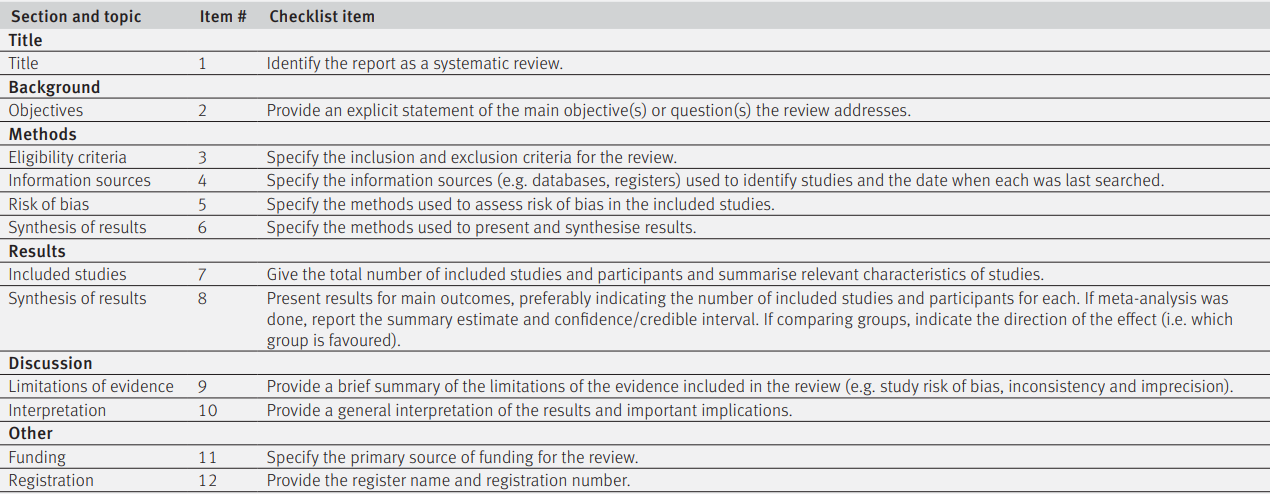
Supplementary Fig. S2. PRISMA 2020 for Abstract checklist** ^1^**.**


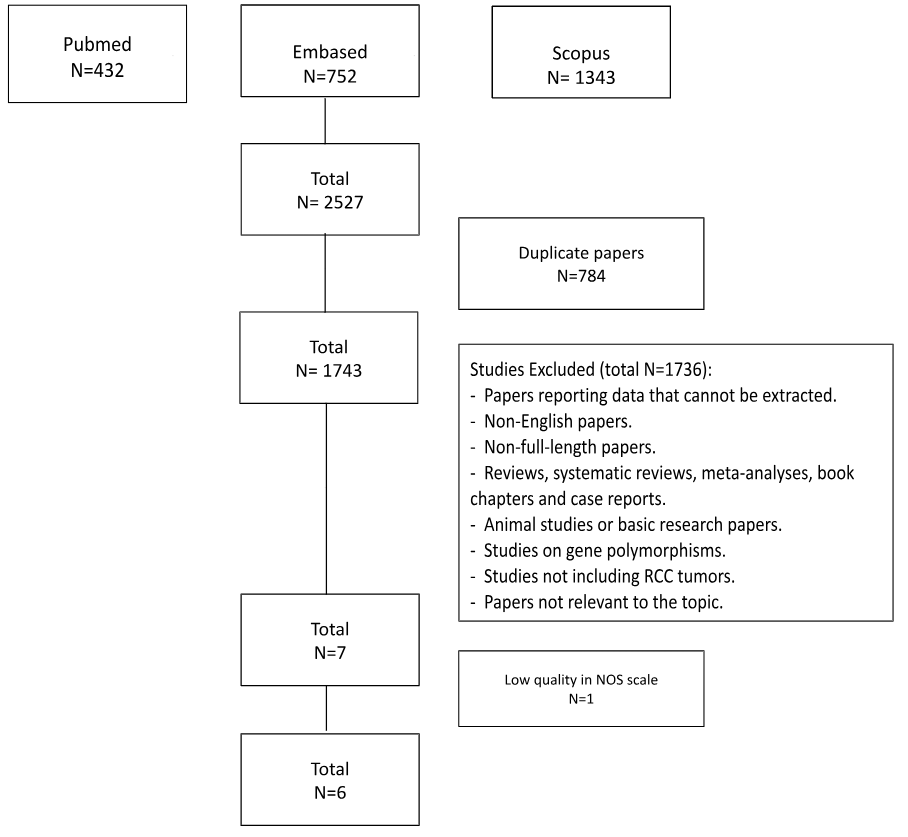
 **Supplementary Fig. S3. PRISMA Flowchart.** PRISMA flowchart of the selection of relevant publications included in the systematic literature review. N indicates the number of papers.


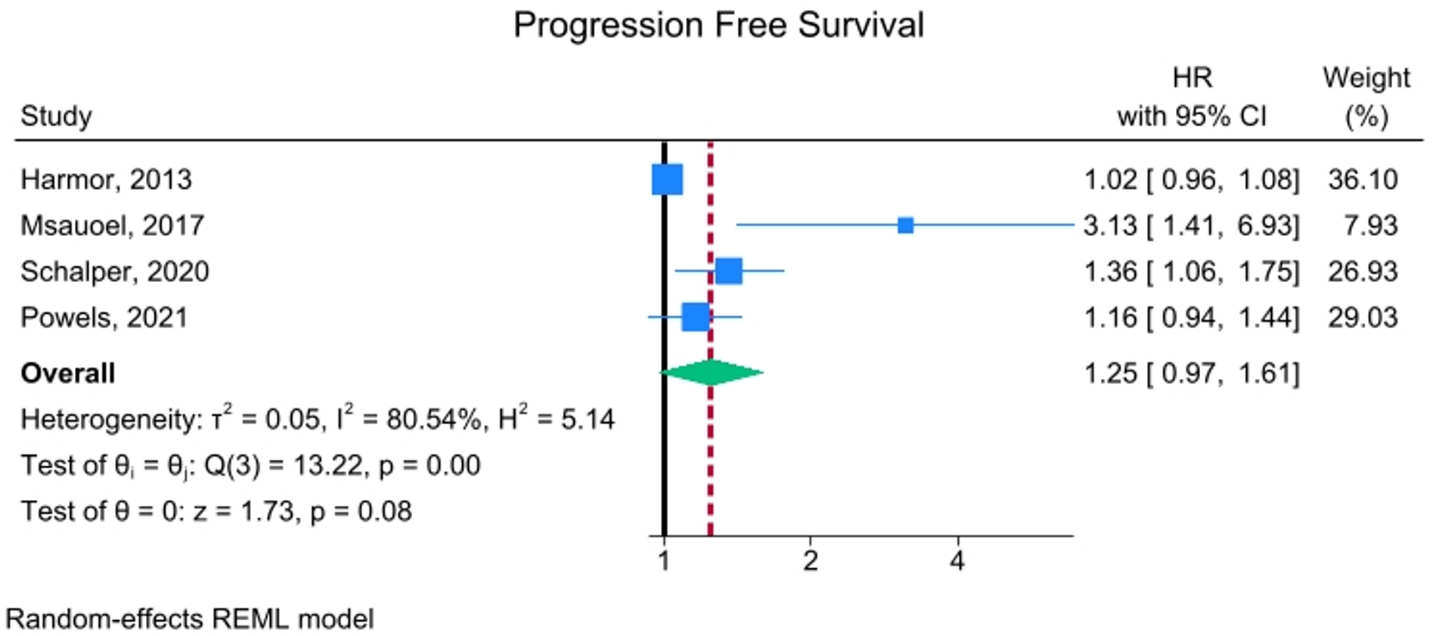


**Supplementary Fig. S4. PFS.** In this figure, the study by Bilen, which provided data from univariable analysis, has been excluded. The analysis shows that this study does not have a significant impact on the results, and its weight is minimal (3.97%).


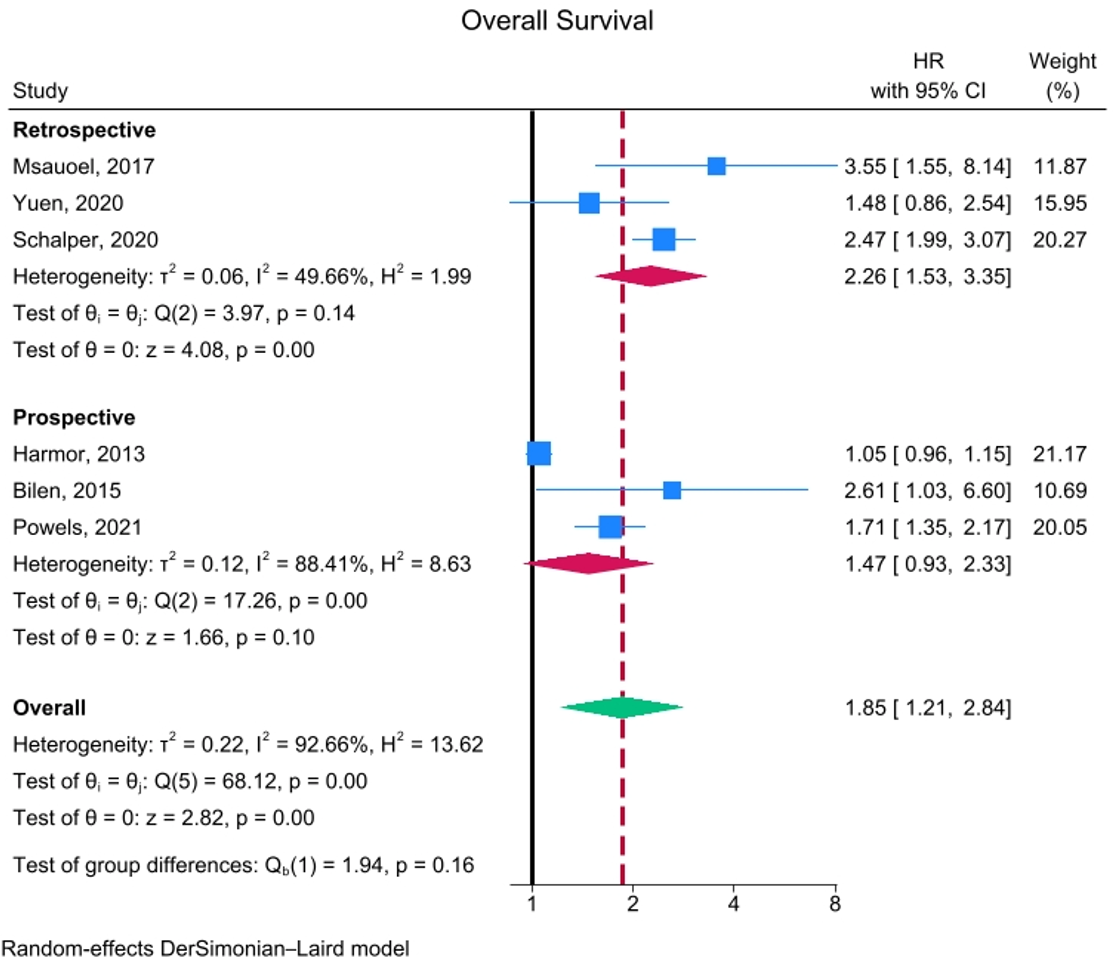


**Supplementary Fig. S5. Subgroup Analysis: OS in Prospective and Retrospective Studies.** Forest plot of HR comparing OS in low and overexpressed IL-8 patients divided by retrospective or prospective studies. HRs for each trial are represented by the squares, and the horizontal line crossing the squares represents the 95 % CI. The diamonds represent the estimated overall effect based on the meta-analysis random effects of the trials. All statistical tests were two-sided.

**
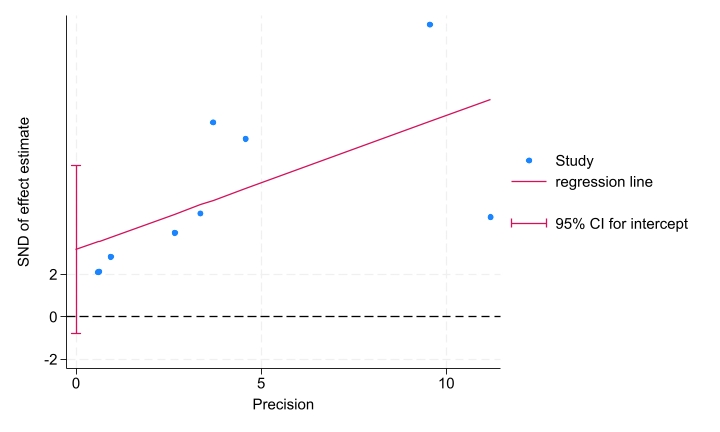

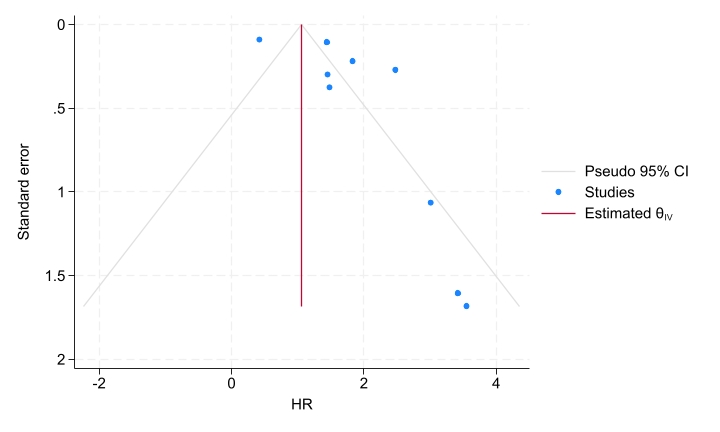
**

**Supplementary Fig. S6. Egger’s test and funnel plot**


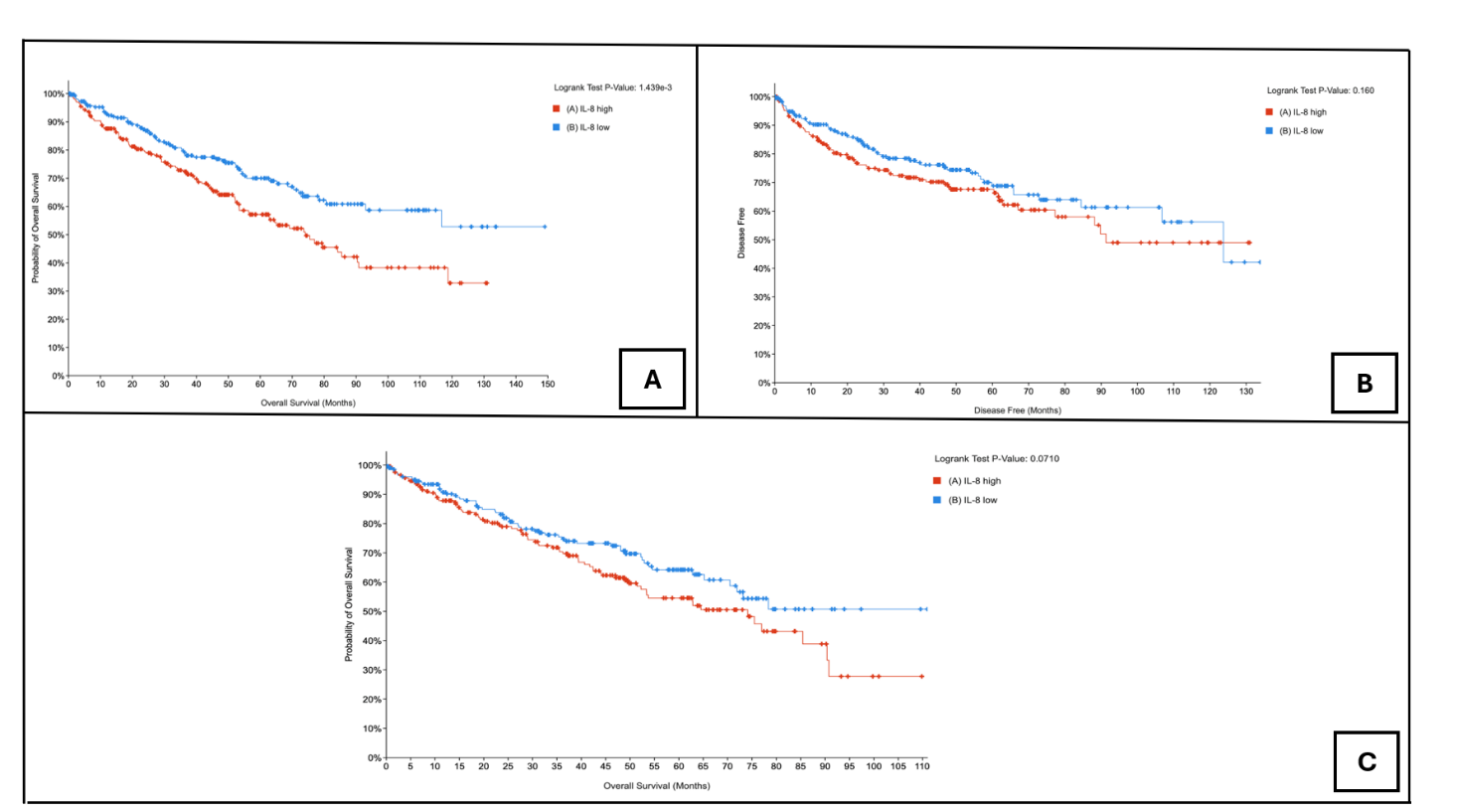


**Supplementary Fig. S7**

**Panel A and B. IL-8 mRNA expression (RNA Seq V2 RSEM) in ccRCC tissue samples (TCGA, Firehose Legacy) and correlation with OS and DFS respectively.**

**Panel C. IL-8 mRNA expression (RNA Seq V2 RSEM) in ccRCC tissue samples (TCGA, Nature 2013) and correlation with OS.**


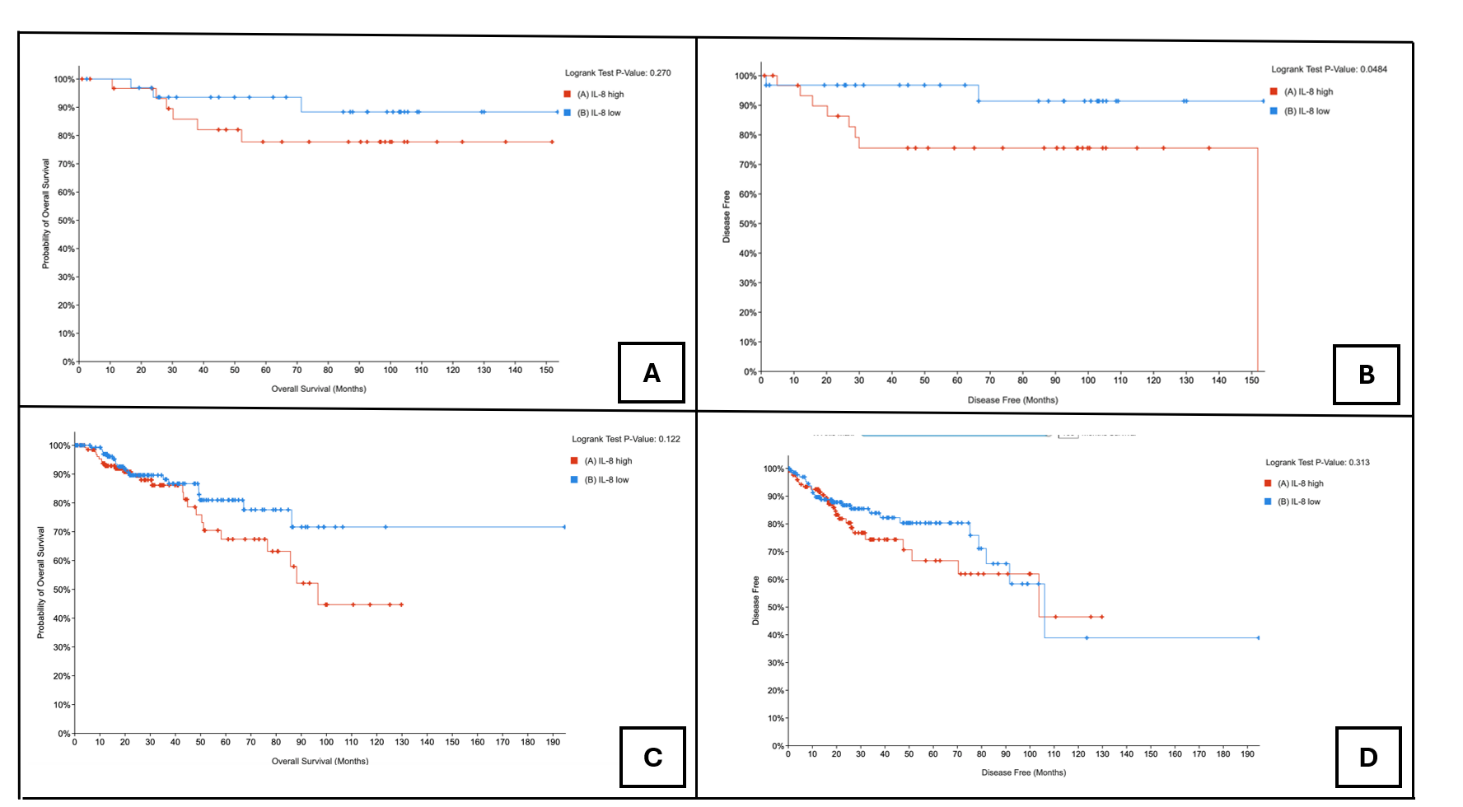


**Supplementary Fig. S8.**

**Panel A and B. IL-8 mRNA expression (RNA Seq V2 RSEM) in chromophobe nccRCC tissue samples (TCGA, Firehose Legacy Chromophobe) and correlation with OS and DFS respectively.**

**Panel C and D. IL-8 mRNA expression (RNA Seq V2 RSEM) in papillary nccRCC tissue samples (TCGA, Firehose Legacy Firehose Legacy Papillary) and correlation with OS and DFS respectively.**


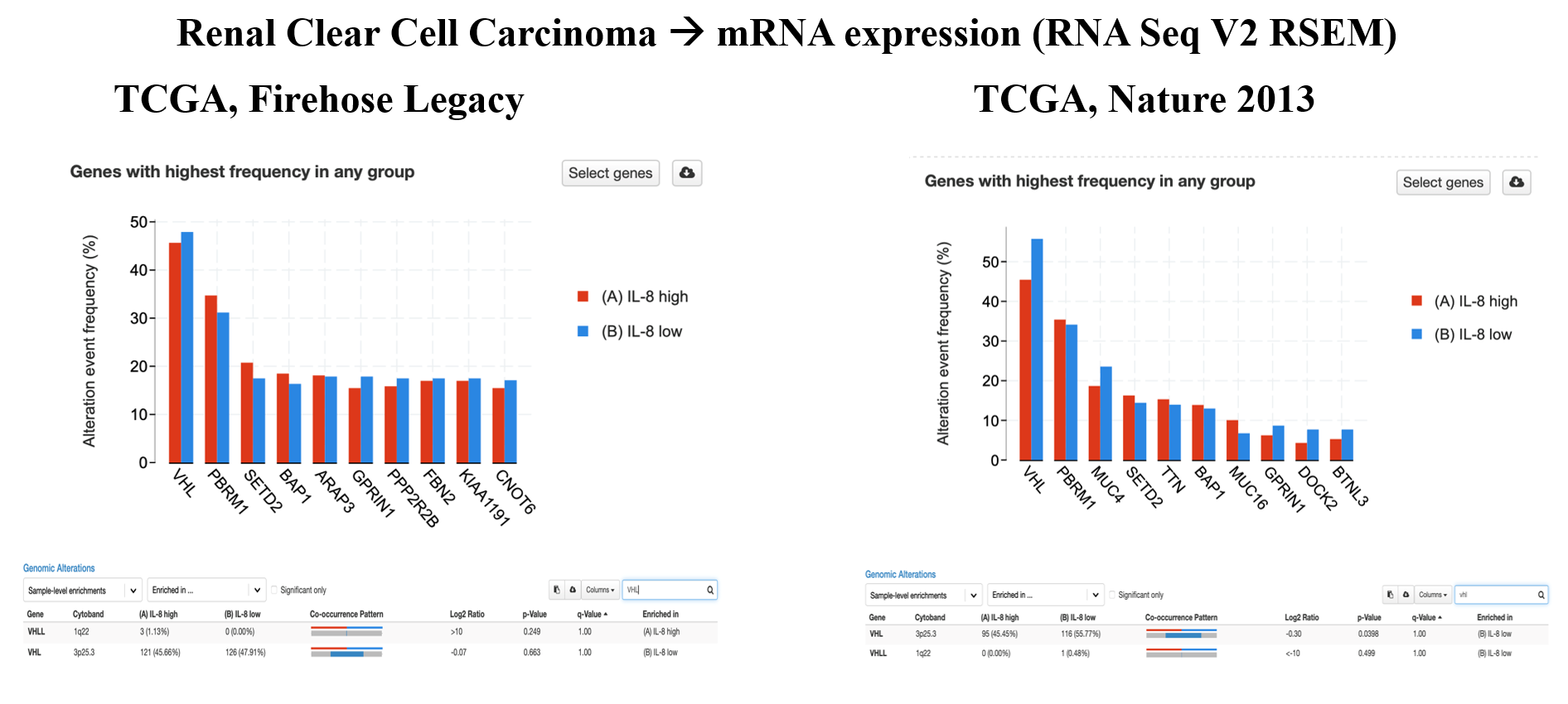
**Supplementary Figure S9.
Left and right panels: Frequently mutated genes in ccRCC tissue samples, stratified by IL-8 high and IL-8 low expression (TCGA, Firehose Legacy; TCGA, Nature 2013).**


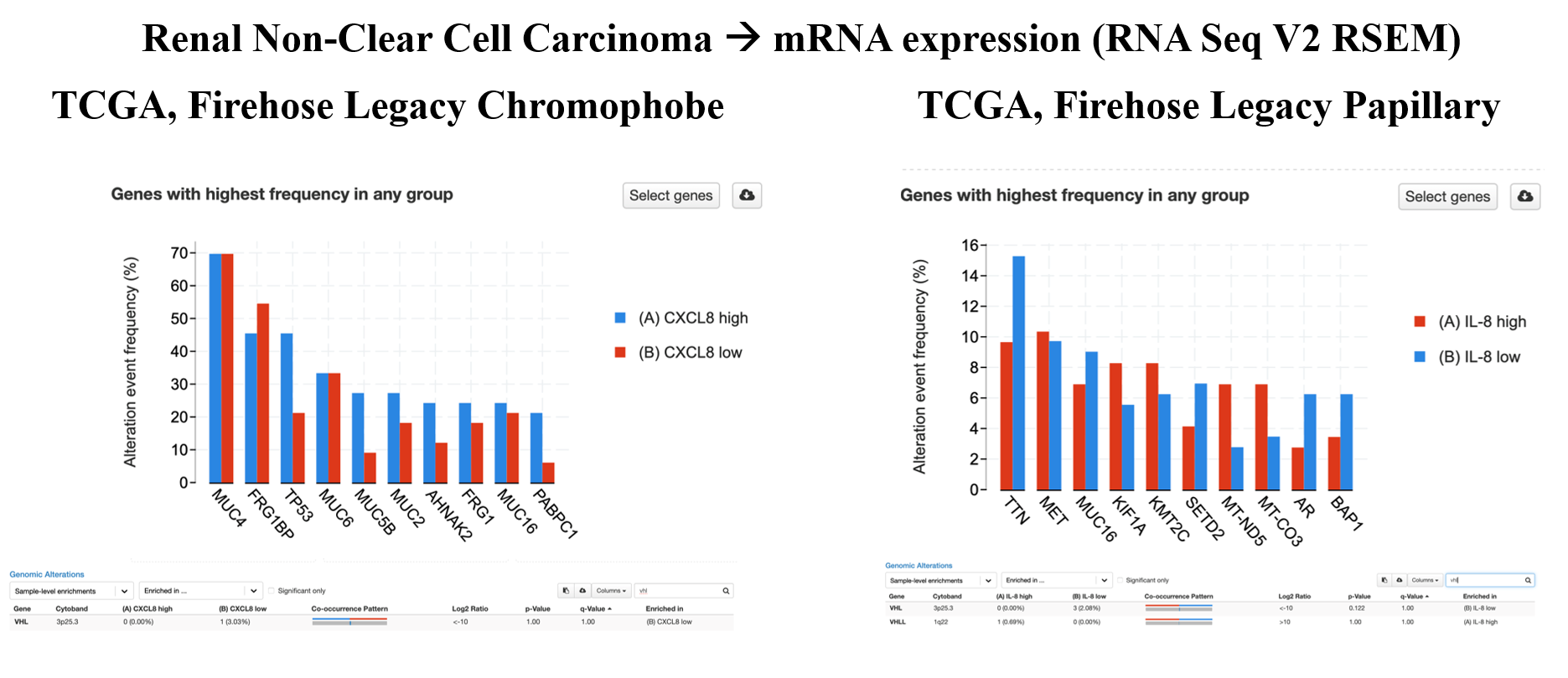


**Supplementary Figure S10.
Left and right panels: Frequently mutated genes in nccRCC tissue samples, stratified by IL-8 high and IL-8 low expression (TCGA, Firehose Legacy Chromophobe; TCGA, Firehose Legacy Papillary).**

| **PubMed:** ("Interleukin-8" OR “IL-8” OR “interleukin8” OR “IL8” OR “CXCL-8” OR “CXCL8” [Mesh]) AND (((“Rena*”) AND (“Carcinoma” OR “Cancer” OR “Tumor” OR “Neoplas*”)) OR (“RCC” OR “pRCC” OR “chRCC” OR “ccRCC”)); |
| --- |
| **Scopus:**  ((( TITLE-ABS-KEY ( "Il-8" ) ) OR ( TITLE-ABS-KEY ( "Il8" )) OR ( TITLE-ABS-KEY ( "Interleukin8" )) OR ( TITLE-ABS-KEY ( "Interleukin-8" )) OR ( TITLE-ABS-KEY ( "CXCL-8" )) OR ( TITLE-ABS-KEY ( "CXCL8" ))) AND ( TITLE-ABS-KEY ( "rena*" ))) AND (( TITLE-ABS-KEY ( "carcinoma" )) OR ( TITLE-ABS-KEY ( "cancer" )) OR ( TITLE-ABS-KEY ( "tumor" )) OR ( TITLE-ABS-KEY ( "neoplas*" ))) OR (( TITLE-ABS-KEY ( "RCC" )) OR (TITLE-ABS-KEY ("ccRCC")) OR (TITLE-ABS-KEY ("chRCC" )) OR (TITLE-ABS-KEY ("pRCC" ))). |
| **Embased:** ‘Interleukin 8’/exp OR ‘Interleukin 8’ OR Interleukin8 OR ‘il 8’/exp OR ‘il 8’ OR il8 OR ‘cxcl 8’ OR cxcl8 AND (((rcc OR pRCC OR ‘chrcc’/exp OR chRCC OR ‘ccrcc’/exp OR ccrcc) OR ((‘renal/exp OR renal) AND (‘carcinoma’/exp OR carcinoma OR ‘cancer’/exp OR cancer OR ‘tumor?/exp or ‘tumor’/exp OR tumor OR ‘neoplasm’/exp OR neoplasm))) AND (2008:py OR 2009:py OR 2010:py OR 2011:py OR 2012:py OR 2013:py OR 2014:py OR 2015:py OR 2016:py OR 2017:py OR 2018:py OR 2019:py OR 2020:py OR 2021:py OR 2022:py OR 2023:py OR 2024:py) AND ('interleukin 8'/dd OR 'pazopanib'/dd OR 'placebo'/dd OR 'sunitinib'/dd) AND ('advanced cancer'/dm OR 'inflammation'/dm OR 'kidney carcinoma'/dm OR 'metastasis'/dm OR 'neoplasm'/dm OR 'renal cell carcinoma'/dm) AND ('case report'/de OR 'clinical article'/de OR 'clinical study'/de OR 'clinical trial'/de OR 'clinical trial topic'/de OR 'cohort analysis'/de OR 'comparative study'/de OR 'control group'/de OR 'controlled clinical trial'/de OR 'controlled study'/de OR 'cross sectional study'/de OR 'diagnostic test accuracy study'/de OR 'human'/de OR 'human cell'/de OR 'human tissue'/de OR 'major clinical study'/de OR 'meta analysis'/de OR 'meta analysis topic'/de OR 'multicenter study'/de OR 'normal human'/de OR 'observational study'/de OR 'phase 1 clinical trial'/de OR 'phase 1 clinical trial topic'/de OR 'phase 2 clinical trial'/de OR 'phase 2 clinical trial topic'/de OR 'phase 3 clinical trial'/de OR 'phase 3 clinical trial topic'/de OR 'pilot study'/de OR 'proportional hazards model'/de OR 'prospective study'/de OR 'randomized controlled trial'/de OR 'randomized controlled trial topic'/de OR 'retrospective study'/de OR 'systematic review'/de OR 'validation process'/de). |

**Supplementary Tab.S1- The complete search strategies conducted in each of the three electronic databases (PubMed, Scopus and Embased) used for the systematic literature search and the metanalysis.**

| **N** | **Study** | **Criterion score** | | | **Total** |
| --- | --- | --- | --- | --- | --- |
|  |  | Selection | Comparability | Outcome |  |
| **1** | Bilen MA, 2015 | *** | ** | *** | 8 |
| **2** | Powel T, 2021 | **** | ** | *** | 9 |
| **3** | Msaouel P, 2017 | *** | ** | *** | 8 |
| **4** | Harmon CS, 2014 | *** | * (inf) | *** | 7 |
| **5** | Yuen KC, 2020 | *** | * | *** | 7 |
| **6** | Schalper KA, 2020 | *** | *(il8) | *** | 7 |

**Supplementary Tab.S2-New Castle Ottawa Scale (NOS) used to evaluate the quality of trials included in the metanalysis reporting OS**^2^**.** The NOS scale scores were assigned by two independent reviewers.

| **N** | **Study** | **Criterion score** | | | **Total** |
| --- | --- | --- | --- | --- | --- |
|  |  | Selection | Comparability | Outcome |  |
| **1** | Bilen MA, 2015 | *** | ** | *** | 8 |
| **2** | Powel T, 2021 | **** | ** | *** | 9 |
| **3** | Msaouel P, 2017 | *** | ** | *** | 8 |
| **4** | Harmon CS, 2014 | *** | * (inf) | *** | 7 |
| **5** | Schalper KA, 2020 | *** | *(il8) | *** | 7 |

**Supplementary Tab.S3-New Castle Ottawa Scale (NOS) used to evaluate the quality of trials included in the metanalysis reporting PFS**^2^**.** The NOS scale scores were assigned by two independent reviewers.

**Supplementary References**

1. *Page MJ,et al. The PRISMA 2020 statement: an updated guideline for reporting systematic reviews. BMJ. 2021 Mar 29;372:n71. doi: 10.1136/bmj.n71. PMID: 33782057; PMCID: PMC8005924.* 2. Wells GA, Shea B, O'Connell D, Peterson J, Welch V, Losos M, et al. The Newcastle-Ottawa Scale (NOS) for assessing the quality of nonrandomized studies in meta-analyses. Available at: http://www.ohri.ca/programs/clinical_epidemiology/oxford.htm.
